# Impact of simulated cigarette excise tax increase on its consumption in Iran

**DOI:** 10.4178/epih.e2020054

**Published:** 2020-07-23

**Authors:** Behzad Raei, Sara Emamgholipour, Amirhossein Takian, Mehdi Yaseri, Ghahreman Abdoli

**Affiliations:** 1 Department of Health Management and Economics, School of Public Health, Tehran University of Medical Sciences, Tehran, Iran; 2 Health Equity Research Centre (HERC), Tehran University of Medical Sciences, Tehran, Iran; 3 Department of Epidemiology and Biostatistics, School of Public Health, Tehran University of Medical Sciences, Tehran, Iran; 4 Faculty of Economics, University of Tehran, Tehran, Iran

**Keywords:** Smoking, Cigarette, Taxes, Elasticity, Iran

## Abstract

**OBJECTIVES:**

To assess the impact of a simulated tax-induced cigarette price increase on its consumption by different expenditure clusters in Iran.

**METHODS:**

Employing consecutive cross sections for cigarette consumption, a two-part model was applied for different expenditure groups.

**RESULTS:**

A 75% price increase in cigarettes noticeably— as is common in some countries with strong tobacco control policies—reduces current consumption in all five social classes, causing nearly 8% of current male smokers to quit or not to start.

**CONCLUSIONS:**

Findings of the current study suggest that Iranian policy makers go through to implement tobacco taxation policies to control smoking prevalence, which in turn might lead to a reduction in national healthcare expenditures as well as enhance the global community’s capacity to meet Sustainable Development Goals.

## INTRODUCTION

Tobacco use is a leading risk factor for premature death and chronic disability [1]. According to the World Health Organization (WHO) estimate, approximately 7 million preventable deaths are annually attributed to smoking worldwide, with 80% of them occurring in low-income and middle-income countries (LMICs) [2]. If current smoking uptake trends persist, the number of tobacco-related deaths will amount to 8.3 million by 2030, claiming more lives than acquired immune deficiency syndrome, suicide, homicide, automobile accidents, tuberculosis and maternal mortality combined in Asia [3].

Currently, 1.4 billion people aged 15 years and above are tobacco user in the world, 1.12 billion of them are male and 279 million are female. There is a relative reduction in global smoking rates, as well as the actual number of smokers has decreased from 1.46 billion in 2007 to 1.4 billion in 2019, showing little change [4]. To combat the global epidemic of tobacco, the WHO Framework Convention on Tobacco Control (FCTC) in 2003 was adapted and signed by 181 countries as of May 2018, requiring parties to implement some measures to reduce the consumption of tobacco [5]. The WHO introduced the six (monitor, protect, offer, warn, and enforce, and raise; MPOWER) strategies in line with the FCTC to support and accelerate implementation of a wide range of tobacco control policies [4]. The “R” measure of MPOWER focuses on raising taxes on tobacco products, so that tax as a most effective tobacco control measure should be at least 70% of retail sales price [6]. This intervention may play a major part in achieving the target of 30% reduction in the prevalence of adult tobacco use by 2025 based on a 2010 baseline, set by WHO, and paves the way for attaining the sustainable development goal of lowering deaths attributable to non-communicable diseases (NCDs) by 30% by the year 2030 [7]. There is evidence from trends in tobacco smoking suggesting only 24 out of 146 countries are on track to reach 30% relative reduction in tobacco smoking prevalence by 2025 [8]. Using taxes to increase the price of tobacco products in LMICs has been recognized to be the most cost-effective anti-smoking intervention [9]. Estimates of the price elasticity of demand for tobacco by the World Bank indicated that a 10% increase in price will reduce the consumption by 4% in high-income countries and by about 8% in LMICs, so that it appears that developing countries are more price sensitive than developed ones [10]. Iran is among the countries where prices increased above tax rise, resulting in a fall in tax share for a specific or mixed excise structure and the total tax share as a percentage of the retail price is only 21.7% [4]. Despite an upward trend in real prices, cigarettes are getting markedly affordable in most developing countries: over the past decade there has been a price reduction of 9.0% and 9.1% in local and international brands, respectively [11]. According to Nemati et al. [12], cigarette is the most-used form of tobacco consumption in Iran and its prevalence was estimated 20.2% in males and 0.8% in females, indicating that smoking rates have been rising steadily. Annually, there were an estimated 50,000 deaths in the country attributable to smoking and this figure is expected to grow, reaching 200,000 cases a year over the coming ten years [13]. Hence, a gradual escalation in the prevalence of smoking is expected in Iran, unless effective initiatives are implemented to curb cigarette use. Raising price of tobacco products through taxation is arguably the most effective instrument for smoking cessation and against smoking initiation and consumption [14]. In Iran, taxes comprise a tiny amount of the price of cigarettes. This is relatively low compared to many countries. This research is to explore the potential for reducing cigarette smoking rates through increases in excise taxes, a public policy that might give rise to permanent reductions in smoking. We will examine the effects of a price increase of 25%, 50%, and 75% to examine the number of smokers who quit or do not start in the different five social classes.

## MATERIALS AND METHODS

The data source for our study is the Households Income and Expenditure Survey (HIES) for the time period 2001 to 2017. The HIES is administered by the Statistical Centre of Iran (SCI) and is representative at the national level. SCI selects sample households based on a three-stage stratified sampling method. In the first step, sample cities called strata are selected based on certain criteria from all provinces nationwide. Then, within selected cities, residential districts are delimitated and drawn using systematic sampling. Finally, sample households are randomly selected from each residential district. Household data are compiled by interview, registration and observation methods. Each interviewer records the data on consumption expenditures and income of samples in the household budget survey questionnaire.

The HIES procedures specify that the individual interviewed can be the head of the household, spouse or partner, or a household member at least 15 years old (the datasets, manuals, questionnaires and related documentation for the 2001 to 2017 SCI surveys are available at https://www.amar.org.ir/default.aspx). A total sample of 570,988 households from 2001 to 2017 were included for analysis in our study. The Stata version 15.1 (StataCorp., College Station, TX, USA) is used to perform the relevant analysis and estimates.

### Cigarette demand by different income groups

Because taxation policies appear to have different effect on smoking behavior, demand equations were estimated across the five income (proxied by expenditure) groups independently. Each group is considered as a distinct subsample of the population, which allows us to distinguish between responses of smokers to price increases, separately. Among 570,988 households, 21.2% have at least one smoker who consumed an average of 16.2 cigarettes per day (Table 1). Our calculation illustrated that the smoking rates and number of cigarette sticks consumed per day peaked for the 2nd and 3rd quintiles in the country. In addition, the poorer quintiles are spending higher share of their income on cigarette. Quintiles were defined by the distribution of households in terms of expenditure per equivalent adult.

The percentage of households that smoked and the number of sticks smoked by smoker households reached a peak among middle economic status (Table 1). The percentage of household income spent on cigarettes declines, however, as households become wealthier.

### Models

In the current study, the 2-part estimation procedure is used to model the parameters associated with cigarette smoking behaviors. This is a 2-stage approach, the first stage is a probit model to address individual’s participation decisions (a dichotomous choice model), and the second one employed an ordinary least squares (OLS) regression to examine quantity of demand (a contribution level model) on condition that the choice was made in the first stage [15]. In the first stage, the dependent variable is a dummy equal to 1 if the individuals observation includes contribution to an activity of interest, and 0 otherwise. So, the first stage attempts to determine the factors that explain the decision of whether or not to contribute while stage 2 is limited to those consumers having contribution. The two-part model can be expressed as follows:

(1) $$\left.\begin{array}{r} Z_{i}^{*}\;=\;W_{i}^{'}a\;+\;e_{i}\\ Z_{i}\;=\;0\;if\;Z_{i}^{*}\;\leq \;0\\ Z_{i}\;=\;1\;if\;Z_{i}^{*}\;>\;0 \end{array}\right\}\;\mathrm{parametric}\;\mathrm{binary}\;\mathrm{probability}\;\mathrm{model}\;(\mathrm{part}\;\mathrm{one})$$

(2) $$\left.\begin{array}{r} y_{i}^{*}\;=\;X_{i}^{'}\beta \;+\;\mu_{i}\;y_{i}=\;y_{i}^{*}\;if\;Z_{i}\;=\;1\\ y_{i}\;not\;observed\;Z_{i}\;=\;0 \end{array}\right\}\;\mathrm{Linear}\;\mathrm{model}\;(\mathrm{part}\;\mathrm{two})\;$$

Where, *Z_i_* is a dichotomous variable which detects whether or not y is observed, *y_i_* being observed only when *Z_i_*= 1, *W_i_* denotes observable features including the overlapping variables

With *X_i_*, and *α* denotes the vectors of parameters to be estimated. In the linear model, *y_i_* denotes the dependent variable, *X_i_* denotes the observable features of the independent variables, *β* is the parameters to be estimated and *μ_i_* is a normally distributed error term with a mean of zero and a standard deviation σ to be estimated [16].

### Model specification

The probit and linear regression models were used to estimate the elasticity of cigarette demand separately for all expenditure quintiles. These two models yield, respectively, a smoking participation price elasticity and a conditional demand price elasticity. The overall price elasticity is equal to summing together the elasticity of participation and the elasticity of consumption [17].

(3) $$\eta_{t}\;=\;\eta_{p}\;+\;\eta_{c}$$

In which *η_t_* is the overall elasticity, *η_p_* is the participation elasticity, and *η_c_* is the conditional elasticity.

It should be noted that since ratification of the WHO FCTC on November 6, 2005, Iran has shown poor compliance to the FCTC’s raise (MPOWER) measure [18,19]. For example, according to the WHO report on the global tobacco epidemic in 2019 tobacco taxes in Iran remain low, contributing only 21.7% of final cigarette prices, and nationwide cessation programs as well as mass media are yet minimally implemented [4]. In this analysis we focused merely on price policy (taxing cigarette) as a cost-effective strategy for two reasons: first, data from the WHO Eastern Mediterranean Region suggest that tobacco taxation has been weakly enforced in Iran so far. Second, due to the scarcity of data on other tobacco control policy such as legislation on smoke-free environments, mass media anti-tobacco campaigns, bans on tobacco marketing, health warnings, and so forth, we restricted the cigarette demand estimation into the variables, data are often available, assuming that other tobacco control policy would not be changed by the change of prices and have remained constant. Even a research that simulated the impact of all MPOWER measures consistent with the WHO FCTC in Iran using the *Abridged SimSmoke* model represents that a larger proportion of decreased smoking prevalence would be attributable to tobacco taxation rather than other tobacco policies included in MPOWER package. It has been modeled that Increasing cigarette excise taxes to 75.0% of the retail price alone would reduce smoking prevalence by 27.3% within 5 years [20].

Several variables were used to explain the decision to participation in the cigarette smoking and the decision of how much to contribute to the smoking. Based on similar previous studies [21,22] and the availability of information several household characteristics were included as determinants of a household’s decision to smoke. In addition, the results are obtained after the significant variables are examined in the model, and all of the predictor variables are verified as statistically independent without collinearity before the model is finalized.

A probit model was employed to estimate the probability p that a household will be a current smoker. Table 2 defines the variables applied in part one.

(4) $$p(smoke)\;=\;\alpha_{0\;}+\;\alpha_{1}\;\ln\left(income\right)\;+\;\alpha_{2}\;\log\left(Price\right)\;+\;\alpha_{3}\;(Divorce\;rate)\;\;\;\;\;\;\;\;\;\;\;\;\;\;\;\;\;\;\;\;\;\;\;\;\;+\;\alpha_{4}(Unemployment\;rate)\;+\;\alpha_{5}(share\;of\;members\;aged\;15\;to\;18)\;\;\;\;\;\;\;\;\;\;\;\;\;\;\;\;\;\;\;\;\;\;\;\;+\;\alpha_{6}(share\;of\;members\;aged\;>\;64)\;\;\;\;\;\;\;\;\;\;\;\;\;\;\;\;\;\;\;\;\;\;\;\;+\;\alpha_{7}(share\;of\;male\;members\;aged\;19\;to\;64)\;\;\;\;\;\;\;\;\;\;\;\;\;\;\;\;\;\;\;\;\;\;\;\;+\;\alpha_{8}(share\;of\;female\;members\;aged\;19\;to\;64)\;\;\;\;\;\;\;\;\;\;\;\;\;\;\;\;\;\;\;\;\;\;\;\;+\;\alpha_{9}(share\;of\;jobless\;member)\;+\;\alpha_{10}(share\;of\;primary\;educated)\;\;\;\;\;\;\;\;\;\;\;\;\;\;\;\;\;\;\;\;\;\;\;\;+\;\alpha_{11}(share\;of\;university\;educated)\;\;+\;\alpha_{12}age\;+\;\alpha_{13}gender\;\;$$

With the following variable definitions:

*Smoke* Dummy variable that takes a value of 1 if the household spent any money on cigarettes; *Income* Total monthly household income of employed family members from self-employment and salary by Iranian rials (in natural logarithm); *Divorce rate* A province-level variable representing total divorce rate of province where household residing in; *Unemployment rate* A province-level variable representing total unemployment rate of province where household residing in; *share of members aged 15 to 18* Proportion of household members at age between 15 and 18 to household size; *share of jobless member* Proportion of unemployed household members aged between15 and 64 to household size; *share of primary educated* A variable showing proportion of household with at least one member who had a primary education; *share of university educated* share of household with at least one member who hold a university degree; *Age* Age of the household head; Sex Dummy variable that takes a value of 1 if the head of household is male

In the second part using OLS regression we adopted a linear model to estimate the demand for cigarettes for smoker households only. Specifically, we estimated;

(5) $$Ln(Consumption)\;=\;\beta 0\;+\;\beta 1\;\ln\left(income\right)\;+\;\beta 2\;Ln(Price)\;+\;\beta 3(Divorce\;rate)\;+\;\beta 4(Unemployment\;rate)\;+\beta 5(Education)\;+\;\varepsilon \;\;\;$$

Consumption was calculated as the natural logarithm of the number of cigarette sticks smoked per month by the household. Education was a dummy variable for the educational level of the household head: no education, elementary school, junior high school, senior high school, diploma, bachelor, master and above. No education was the omitted (reference) level in the regression equation. The other variables were the same as above. Logarithmic transformation of the variables enables us to conveniently obtain the estimates of income elasticity directly from *β*1 and price elasticity from *β*2.

### Ethics statement

This study has approved in research ethics committee, approval ID: IR.TUMS.SPH.REC.1398.168.

## RESULTS

**Figure 1.**
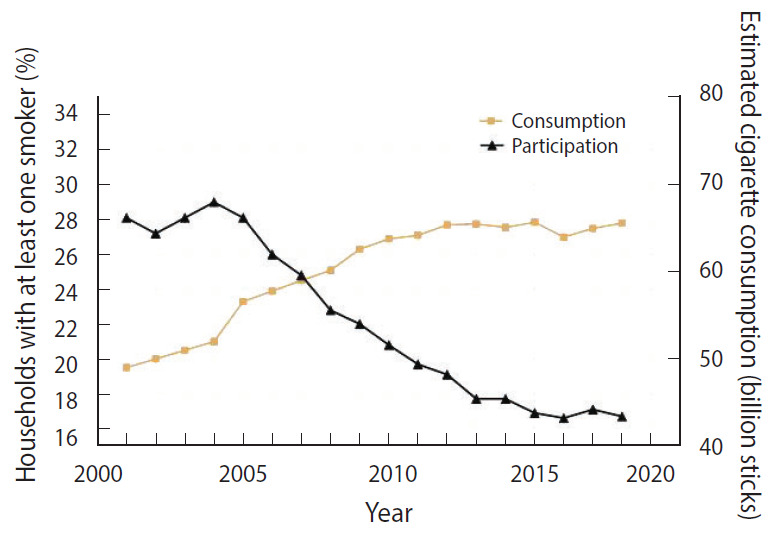
Household smoking prevalence and cigarette consumption.

Overall smoking rates—calculated from the HIES as the percentage of households that have at least one smoker—have decreased slightly over the course of the seventeen survey years, from 28% in 2001 to 16% in 2017 (Figure 1).

Using data from HIES surveys cigarette smoking prevalence rates were calculated and date about the number of cigarette sticks smoked in Iran are released by Ministry of Industry, Mine and Trade every year. As shown in Figure 1, while a decreasing trend is being seen in smoking prevalence, there have been large increases in number of smoked cigarette sticks which might be accounted for by population growth.

The following equation was applied to estimate the price elasticity of participation (EP) [23]:

(6) $$EP\;=\;\frac{\beta_{i}}{\sqrt{2\pi }}exp\;[-\frac{1}{2}{\left(\beta^{'}\bar{X}\right)}^{2}]\frac{1}{E(Y|X)}$$

Both the participation and consumption equations are estimated separately for 5 expenditure quintiles. Tables 2 and 3 present the factors explaining decisions for smoking, estimated by probit and OLS, respectively.

The main outcomes to be drawn from the estimated demand equations are as follows:

(1) The level of education is a deciding factor of the demand for the number of cigarettes, the higher education level is connected with the fewer number of cigarettes consumed. Even for the households with the greater share of members with university degree, there is a less demand for cigarette; (2) Price has a negative effect on both smoking participation and on the number of cigarettes demanded by smokers, with elasticities ranging between -0.43 and -0.49. These results show that increasing cigarette taxes will reduce both smoking participation and consumption; (3) Income coefficients suggest that it slightly influences the likelihood of smoking participation or cessation. Moreover, we noted the significant and positive coefficient for income among smokers, indicating increased income has a positive but negligible impact on the number of cigarettes demanded; (4) As expected, age and sex structure are also significantly correlated with smoking rates. The significant and positive coefficient for the number of male adults living in the household shows that the likelihood of having a smoker in the household increases as the number of male adults in the household increases. In contrast, the number of female adults reduces the likelihood of household participation in smoking.

Table 4 summarizes the price and income elasticities implied by the regression results.

The total price elasticity was computed by summing the elasticities from the first and the second part of the estimation. This reveals that a 10% increase in the cigarette price would result in a 4.6% decrease in average cigarette consumption, ceteris paribus. According to this finding, cigarette consumption is more sensitive to price changes than smoking participation.

Overall income elasticity shows that cigarette demand appears to be rather inelastic with income elasticity close to zero.

### Simulations

We applied a simple and static model to estimate the effect of three cigarette price hike scenarios (25, 50, and 75%) on the current number of smokers, stratified by expenditure quintiles. No changes in cigarette consumption patterns were assumed as the baseline scenario. we also assumed that taxes will be adjusted for inflation annually to maintain the same proportional level above the price. For each expenditure group, the number of quitters owing to a price increase is a product of the (1) actual number of smokers in that expenditure group, (2) participation elasticity of cigarette demand, (3) and actual magnitude of the price increase. Iran male population aged 15 and more was classified in to five income groups using data on socioeconomic status released by SCI in 2018, then the number of smokers in each quintile was calculated by multiplying the number of individuals in each group in smoking prevalence rate of the same group.

Table 5 presents the result of the counterfactual scenarios based on the participation-price elasticity analysis. In this projection, it was assumed that cigarette prevalence among households is equivalent to that among male aged 15 years and over. This assumption mirrors the findings of a great deal of the previous work in this field. A 75% price increase in cigarettes noticeably— as is the common in some countries with strong tobacco control policies—reduces current consumption in all five social classes, causing nearly 8% of current male smokers to quit or not to start. Given that quintile 3 accounts for the higher proportion of the total population with maximum prevalence of smoking, the reduction in smoking is strongly concentrated in this class. If the excise tax were to be set at 50%, the prevalence of cigarette smoking among men would be dropped by 5%.

## DISCUSSION

Since Iranian government signed FCTC on June 16, 2003, the Iranian Anti-Tobacco Association in conjunction with the Iranian Ministry of Health and Medical Education made nascent but important, concrete steps towards a comprehensive national tobacco control law. With great effort, the FCTC was ratified by the Iranian Parliament in the form of 20 Articles and 3 Notes and the law was enacted in line with the goals of FCTC to combat tobacco epidemics and protect public health. This law imposed new requirements on tobacco control efforts including smoke-free environments, mass media anti-tobacco campaigns, bans on tobacco marketing, health warnings, cessation of treatment for tobacco dependence, and restrictions on access for youth. In spite of the fact that the law is a very restrictive on paper, it still has not been fully implemented in reality [24]. In terms of its execution, numerous articles of this law have been neglected by both the Iranian government and Iranian consumers. According to the WHO estimates, Iran will not achieve the smoking component of the global NCD target if effective and sustained action is not supported [19]. Data from the WHO Eastern Mediterranean Region suggest that tobacco taxation has been weakly enforced in Iran so far and taxes as a percentage of retail price were at the lowest level, totaling only 21.7% of the retail prices and there is ample room to raise tobacco taxes. Our empirical results have indicated (1) that the price elasticity of demand for cigarettes is in the range of -0.43 to -0.49, (2) price effects appear to be lowest for the richest quintile, and (3) that income changes have a negligible effect on cigarette demand. Based on the findings, no obvious pattern in terms of magnitude of price elasticity across the classes was observed. The results of the current study are consistent with those of Bahram et al. [25] who found that there is no consistent price responsiveness between income deciles one and ten. We noticed that for the five expenditure clusters, increases in cigarette prices (in the range of 25–75%) effectively reduce the number of smokers. Our price-elasticity estimates produce one interesting piece of evidence for policy decision and evaluation. One of the issues emerging from these findings is that price elasticity is lowest in the top expenditure group. This finding is in agreement with Salti et al. [21] who found that richest group is slightly less price responsive than the other quintiles. What is noteworthy is that taxation policies must be focused on raising the real price, unless cigarette becomes more affordable as incomes and prices of other commodities go up [26]. Our estimate indicates the all expenditure clusters are income inelastic, not following a regular pattern. These results have implications for any future attempts by the government of Iran to impact on cigarette demand by means of taxation policy. The long-run impact of tax increases would be noticeable. For instance, if the tax increases were completely passed on to the consumer, and the current excise tax would triple to 75% of retail prices, cigarette demand would decrease by about 34%. The fall-off in demand would arise from a rough estimate of 8% decline in smoking participation and 26% decline in the quantity of cigarettes smoked by smokers. In line with other previous studies, this research found unemployment rate is associated with smoking intensity but divorce rate is not significantly correlated [27,28]. These results suggest that raising cigarette prices through taxation is an effective tool to reduce smoking prevalence for all income groups, but the magnitude of the effects would differ across these groups. We do not explicate here the dilemma of smuggling of cigarette. It deserves mentioning that Iran are among target markets for cigarette smuggling, where open sale of tax-free illegal cigarettes is causing a great taxation loss to the government [29]. Nevertheless, studies have reported that moderate smuggling due to a high transaction cost fails to dilute the influence of higher excise tax prices and retail price of black -market cigarettes increases, as well [30]. In this study, the relatively low-price elasticities are due, in part, to increasing affordability of cigarettes in the country. Only do non-price interventions not suffice to reduce smoking prevalence by the 30% in LMICs by 2025, but they are needed to be coupled with pricing policies. Tripling tobacco taxes has been recommended the most plausible way to do so [31]. Our price elasticity estimates for cigarette was similar to those found in previous literature, which suggested that demand for cigarette are less elastic [32,33]. Economic aspects are one of the most important prevention strategies for smoking cessation and against smoking initiation and consumption in particular in young and teenage smokers who are new starter [34]. A price freeze on cigarettes at any level, whether high or low, is unlikely to encourages consumers to switch their smoking behavior, ceteris paribus. In response to a fall in cigarette sales following price increases, tobacco companies might formulate strategies such as lobbying efforts, discount schemes, price-reducing marketing to weaken the impact of tobacco excise tax increases [30]. Success in controlling tobacco consumption using taxation policy depends to a large extent on after-tax affordability of tobacco products, and the degree of price gap among them [35]. Our simple model suggests that large increases in cigarette prices, if maintained in real terms, are an effective instrument in reducing smoking prevalence. Taken together, all five expenditure groups respond to cigarette price increases by reducing their total cigarette demand. Reducing the gap between expensive and cheap cigarette brands, coupled with taxing cigarettes can be a rigorous, and structured advice to governments of Iran for combatting the epidemic of cigarette smoking.

Several limitations of our study are worth mentioning. First the estimates were based on pooled consecutive cross-sectional data, so our analysis may not demonstrate the long-run price effects. Second, we employed household or individual-level data taken from surveys which were based on self-report and might be subject to recall bias. Third, cigarettes in Iran also have wide variation in prices, allowing consumers to switch to lower–priced brands when taxes increase. Due to the lack of data we could not examine how changes in relative prices may lead to substitution effect in consumer behavior. However, additional research is needed for quantifying the magnitude of switching effect and which smokers are more likely to switch to enact an optimum level of taxation. Fourth, excise taxes will only have an effect when increases are passed onto the consumers through higher retail prices [4], which is what our analysis assumed. If the tax increase were not fully (but partially) passed onto the consumers, the effect of taxing tobacco would be undermined. Moreover, since the HIES is a household-level survey we can compute elasticity for the overall household only, and not for individual household members. Unfortunately, the lack of clear evidence for MPOWER measures across socioeconomic groups precluded us from examining whether such policies could ramp up the fall-off in cigarette smoking. Finally, in this study there were no data on which could be entered into the regression models, as a result, our analysis studied one policy, and thus did not model the effects of all MPOWER policies simultaneously. Our study had a number of advantages, including having a representative sample of the Iranian population, a large sample size at the national and provincial levels for a 17-year period. This is the first study in Iran examining participation price elasticity for cigarette use to see the effect of price increase on smoking prevalence. Finally, we performed our statistical analysis by expenditure groups to look into the gradient in smoking patterns.

The current study indicates that price is a statistically significant contributor in households’ decisions to participate in smoking— and also in decisions about the quantity of cigarettes to smoke. Each 10% increase in the price of cigarettes results in a 5% decrease in cigarette consumption. Household income, in general, has a positive, significant impact on the number of cigarettes consumed while increased income for all quintiles except for Q1 would reduce the probability of a household participation in cigarette smoking. Our simulations represent that —by holding other factors constant and assuming that taxes fully passed onto consumers— a 75% price increase in cigarettes as proposed by WHO would make an important contribution to the reduction in smoking prevalence in Iran. Findings of the current study suggest that Iranian policy makers go through to implement tobacco taxation policies to control smoking prevalence, which in turn might lead to a reduction in national health care expenditures as well as enhance the global community’s capacity to meet Sustainable Development Goals.

## Figures and Tables

**Table 1.** Descriptive statistics of sample households and cigarette use^1^

Variables	Household socioeconomic status, quintile				
	First (poorest)	Second	Third	Fourth	Fifth (richest)
Sample size (household)	114,395	114,455	114,440	114,367	113,331
Average expenditure per equivalent adult (Iranian rial)	1,754,994	3,003,238	4,061,518	5,412,067	9,440,985
Households with male aged between 19 and 64 (%)	20.4	26.4	29.1	31.1	33.2
Households with female aged between 19 and 64 (%)	27.5	30.0	31.0	32.1	33.4
Households that smoked (%)	16.1	20.4	21.5	21.3	19.2
Household expenditures spent on cigarette (%)	6.1	4.4	3.3	2.5	1.6
No. of cigarettes smoked per day (stick)	16.0	16.4	16.6	16.5	15.7
Households with at least one member holding university education (%)	4.1	7.4	11.0	16.1	25.7

1 Data from authors’ calculations from Households Income and Expenditure Survey for the years 2001-2017.

**Table 2.** Estimated probit regression model on smoking participation among each expenditure quintile, based on 2001-2017 Households Income and Expenditure Survey data

Variables	Household socioeconomic status, quintile				
	First (poorest)	Second	Third	Fourth	Fifth (richest)
Total (n)	113,331	114, 367	114,440	114,455	114,395
Income	0.031 (0.005)^***^	-0.036 (0.005)^***^	-0.028 (0.005)^***^	-0.047 (0.005)^***^	-0.015 (0.005)^**^
Price	-0.084 (0.002)^***^	-0.086 (0.002)^***^	-0.092 (0.002)^***^	-0.091 (0.002)^***^	-0.099 (0.003)^***^
Divorce rate	0.096 (0.011)^***^	0.053 (0.010)^***^	0.038 (0.009)^***^	0.048 (0.009)^***^	0.025 (0.008)^**^
Unemployment rate	0.005 (0.000)^***^	0.005 (0.000)^***^	0.002 (0.000)^**^	0.001 (0.000)^*^	0.001 (0.000)
Share of members aged 15 to 18	-0.143 (0.039)^***^	-0.069 (0.036)	-0.017 (0.036)	0.141 (0.036)^***^	0.250 (0.036)^***^
Share of members aged>64	-0.180 (0.034)^***^	-0.550 (0.034)^***^	-0.657 (0.035)^***^	-0.623 (0.037)^***^	-0.642 (0.037)^***^
Share of male members aged 19 to 64	0.512 (0.032)^***^	0.398 (0.029)^***^	0.376 (0.029)^***^	0.422 (0.029)^***^	0.439 (0.028)^***^
Share of female members aged 19 to 64	-0.216 (0.034)^***^	-0.399 (0.032)^***^	-0.386 (0.032)^***^	-0.298 (0.032)^***^	-0.198 (0.033)^***^
Share of jobless member	-0.138 (0.024)^***^	-0.140 (0.024)^***^	-0.085 (0.025)^**^	-0.028 (0.024)	0.047 (0.023)^*^
Share of primary educated	0.459 (0.019)^***^	0.211 (0.018)^***^	0.114 (0.018)^***^	0.129 (0.019)^***^	0.022 (0.020)
Share of university educated	-0.162 (0.041)^***^	-0.469 (0.031)^***^	-0.676 (0.028)^***^	-0.681 (0.025)^***^	-0.669 (0.023)^***^
Age	0.001 (0.000)^***^	0.005 (0.000)^***^	0.007 (0.000)^***^	0.008 (0.000)^***^	0.007 (0.000)^***^
Sex	-0.743 (0.019)^***^	-0.691 (0.019)^***^	-0.674 (0.021)^***^	-0.639 (0.022)^***^	-0.550 (0.022)^***^
Constant (α_0_)	-0.222 (0.097)^*^	1.032 (0.094)^***^	1.010 (0.095)^***^	1.178 (0.097)^***^	0.688 (0.095)^***^

Values are presented as coefficient (standard error).

* p<0.05,

** p<0.01,

*** p<0.001.

**Table 3.** Estimated Linear regression model on smoking intensity among each expenditure quintile, based on 2001-2017 Households Income and Expenditure Survey data

Variables	Household socioeconomic status, quintile				
	First (poorest)	Second	Third	Fourth	Fifth (richest)
Total (n)	18,456	26,041	27,436	26,887	25,767
Income	0.039 (0.006)^*^	-0.001 (0.005)	0.006 (0.006)	0.018 (0.006)^***^	0.027 (0.006)^***^
Price	-0.389 (0.013)^***^	-0.356 (0.010)^***^	-0.358 (0.010)^***^	-0.371 (0.010)^***^	-0.320 (0.010)^***^
Divorce rate	-0.001(0.013)	-0.011 (0.010)	-0.002 (0.010)	-0.005 (0.009)	-0.007 (0.009)
Unemployment rate	0.004 (0.001)^**^	0.006 (0.000)^***^	0.005 (0.000)^***^	0.007 (0.000)^***^	0.004 (0.001)^***^
Education (Ref: no education)					
Elementary school	-0.020 (0.013)	-0.040 (0.010)^***^	-0.050 (0.011)^***^	-0.074 (0.013)^***^	-0.069 (0.015)^***^
Junior high school	-0.064 (0.016)^***^	-0.117 (0.013)^***^	-0.160 (0.013)^***^	-0.180 (0.015)^***^	-0.191 (0.017)^***^
Senior high school	-0.162 (0.038)^***^	-0.274 (0.025)^***^	-0.274 (0.021)^***^	-0.304 (0.021)^***^	-0.300 (0.019)^***^
Diploma	-0.217 (0.041)^***^	-0.284 (0.027)^***^	-0.272 (0.022)^***^	-0.363 (0.022)^***^	-0.295 (0.021)^***^
Bachelor	-0.367 (0.101)^**^	-0.360 (0.054)^***^	-0.440 (0.037)^***^	-0.492 (0.027)^***^	-0.490 (0.021)^***^
Master and above	-0.682 (0.387)	-0.579 (0.186)^*^	-0.511 (0.092)^***^	-0.562 (0.069)^***^	-0.485 (0.037)^***^
Constant (β_0_)	7.89 (0.13)^***^	8.44 (0.11)^***^	8.39 (0.11)^***^	8.32 (0.12)^***^	7.92 (0.19)^***^

Values are presented as coefficient (standard error). Ref, reference.

* p<0.05,

** p<0.01,

*** p<0.001.

**Table 4.** Price and income elasticities by expenditure quintile (Q) groups

Elasticities	Q1 (poorest)		Q2		Q3		Q4		Q5 (richest)	
	Price	Income	Price	Income	Price	Income	Price	Income	Price	Income
Participation	-0.07	0.02	-0.11	-0.04	-0.12	-0.03	-0.12	-0.06	-0.11	-0.01
Consumption	-0.38	0.03	-0.35	0.00	-0.35	0.00	-0.37	0.01	-0.32	0.02
Total	-0.46	0.05	-0.46	-0.04	-0.47	-0.03	-0.49	-0.05	-0.43	0.01

**Table 5.** Model projections using price elasticities based on different price increase scenarios

Quintiles	No. of male population (15 ≤age)	Cigarette prevalence (households)	No. of male smokers	Participation elasticity	No. of male smokers who quit following price increase scenario		
					25%	50%	75%
Q1 (lowest)	6,312,000	0.16	1,041,720	0.07	17,709	35,418	53,127
Q2	6,312,000	0.20	1,302,300	0.11	35,162	70,324	105,486
Q3	6,312,000	0.21	1,344,040	0.12	40,321	80,642	120,963
Q4	6,312,000	0.21	1,337,630	0.12	40,129	80,258	120,387
Q5 (richest)	6,312,000	0.19	1,256,450	0.11	33,924	67,848	101,772
Total	31,560,000	-	6,282,140	-	167,245	334,490	501,735
